# A Rare Case of a Middle Mediastinal Mass With Intense and Diffuse Contrast Enhancement

**DOI:** 10.7759/cureus.72023

**Published:** 2024-10-21

**Authors:** Christopher D VonTungeln, Bryton E Perman, Oscar L Llanos, Hanna E Nale, Lester J Layfield

**Affiliations:** 1 Internal Medicine, University of Missouri Healthcare, Columbia, USA; 2 Pulmonary and Critical Care Medicine, University of Missouri School of Medicine, Columbia, USA; 3 Pathology and Laboratory Medicine, University of Missouri School of Medicine, Columbia, USA

**Keywords:** extra-adrenal paraganglioma, incidental mediastinal mass, paragangliomas pheocromocytomas neuroendocrine tumor, paratracheal mass, sdhb mutation

## Abstract

Masses of the mediastinum have a wide differential diagnosis. Paragangliomas are rare neoplasms of neuroendocrine origin. These masses may be either parasympathetic or sympathetic in origin and have variable presentation. Paragangliomas may very rarely form in the mediastinum and make up a small fraction of masses originating in this location. We present a case of a previously healthy 49-year-old female who was incidentally found to have a middle mediastinal mass with intense and diffuse contrast enhancement on computerized tomography (CT) of the chest with contrast which was ultimately determined to be a paraganglioma. This case highlights the importance of consideration of paragangliomas in the differential diagnosis of a mediastinal mass as well as discusses the diagnosis and treatment of these rare tumors.

## Introduction

The classic teaching when evaluating mediastinal masses is to determine the compartment of their location, allowing the formation of a differential diagnosis. Paragangliomas are tumors of neuroendocrine origin that may very rarely present as mediastinal masses, accounting for less than 0.3% of all masses in this location. These tumors have variable presentation and may cause symptoms secondary to catecholamine excess or mass effect [1,2]. Paragangliomas have a higher heritability rate than many other neoplasms [3]. Certain mutations, such as those in the succinate dehydrogenase complex iron sulfur subunit B (SDHB) gene are associated with a more aggressive clinical course [4]. We present a case of a female who was incidentally found to have a middle mediastinal mass with intense and diffuse contrast enhancement on computerized tomography (CT) with contrast of the chest during the workup of a newly diagnosed ovarian lesion which was determined to be a lower paratracheal paraganglioma. This case was presented as a poster at the American College of Chest Physicians (CHEST) conference on October 7, 2024.

## Case presentation

**Figure 1:**
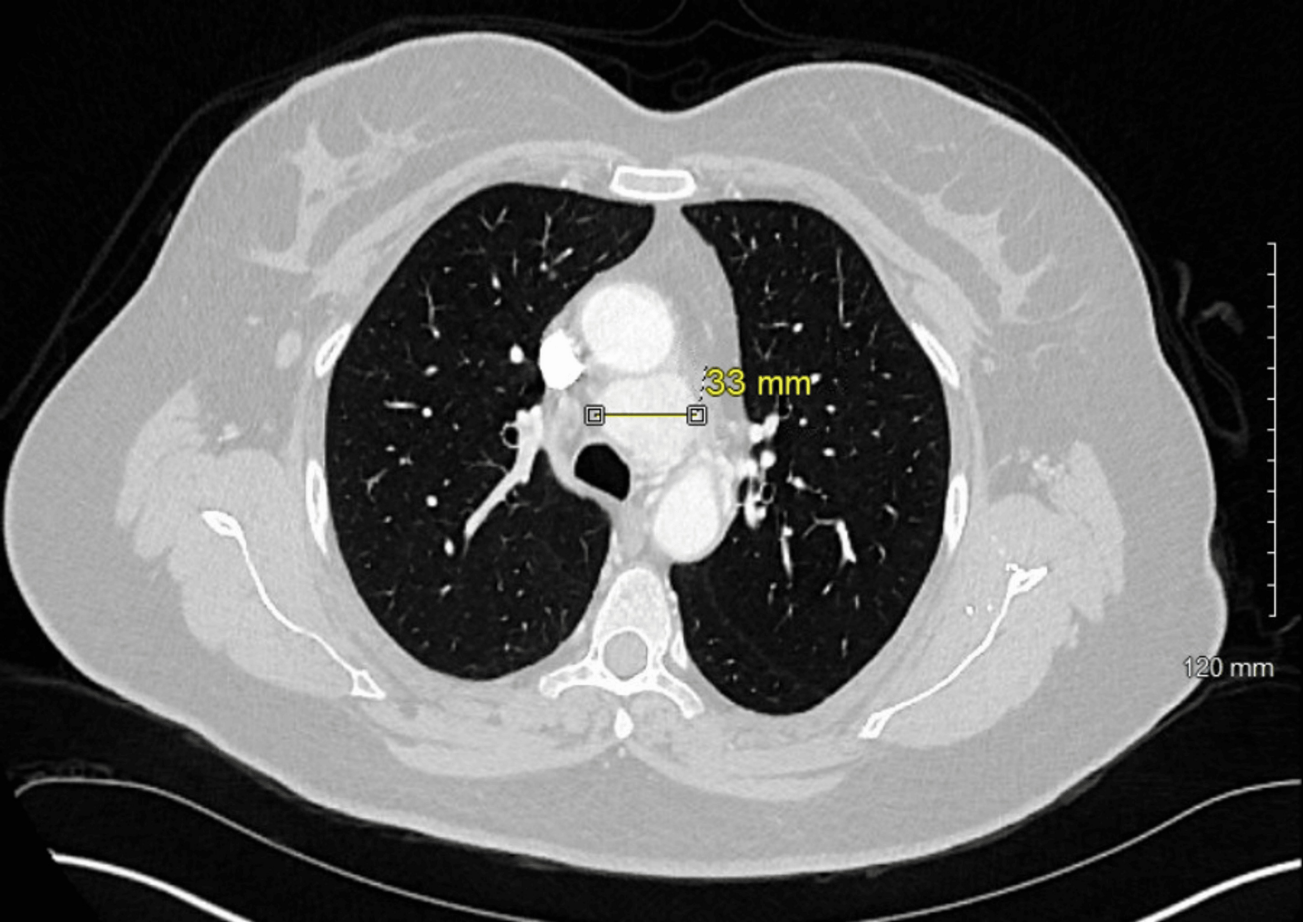
Axial view from CT chest with contrast showing a mediastinal mass with intense and diffuse contrast enhancement

**Figure 2:**
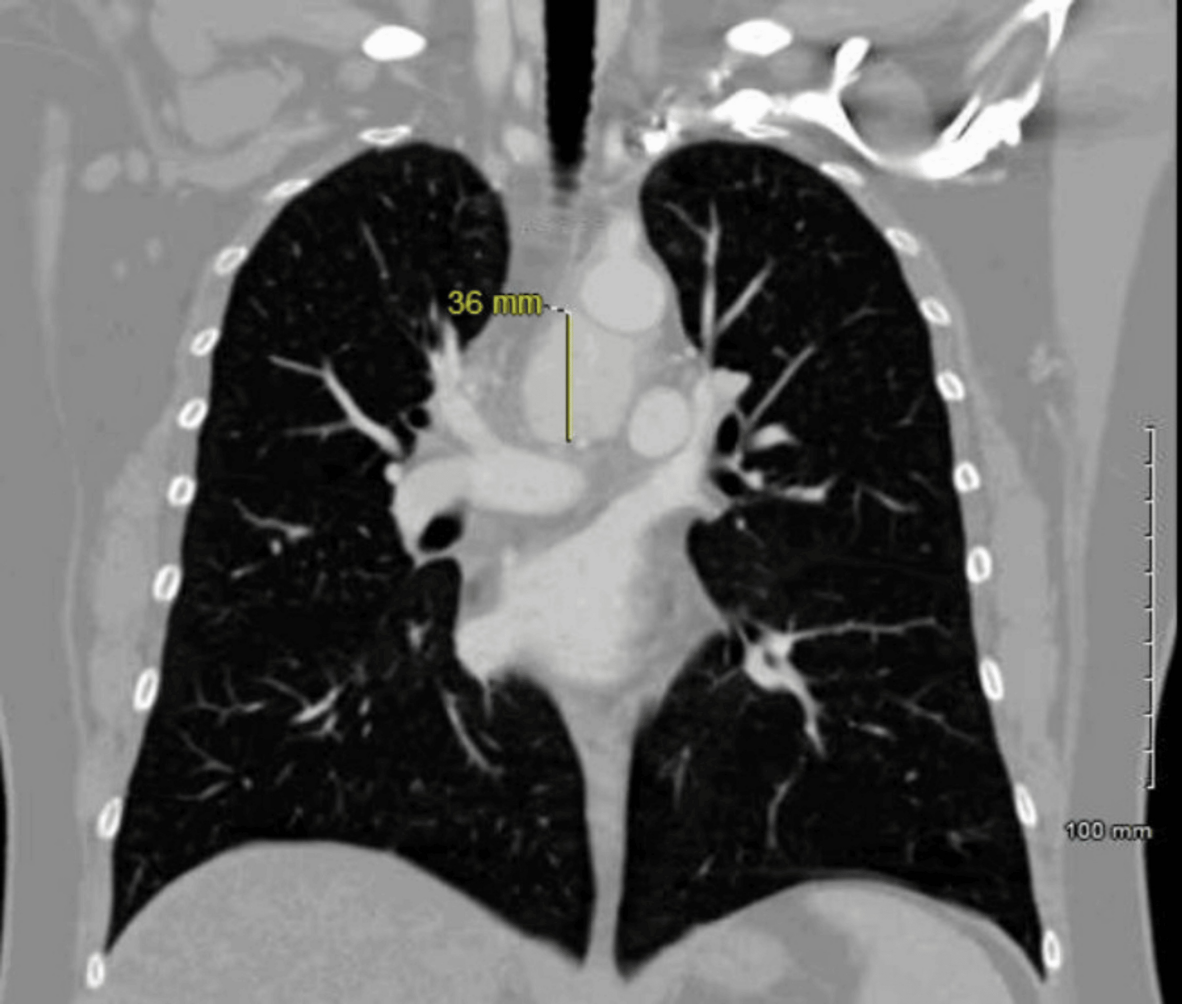
Coronal view from CT chest with contrast showing a hyper-enhancing mediastinal mass

A 49-year-old otherwise healthy female with a recently discovered ovarian mass underwent computerized tomography (CT) of the chest with contrast for preoperative evaluation. The CT showed a lower paratracheal mass measuring 2.8 x 3.3 x 3.6 cm in the maximum axial and craniocaudal dimensions respectively with intense and diffuse contrast enhancement (Figures 1, 2).

**Figure 3:**
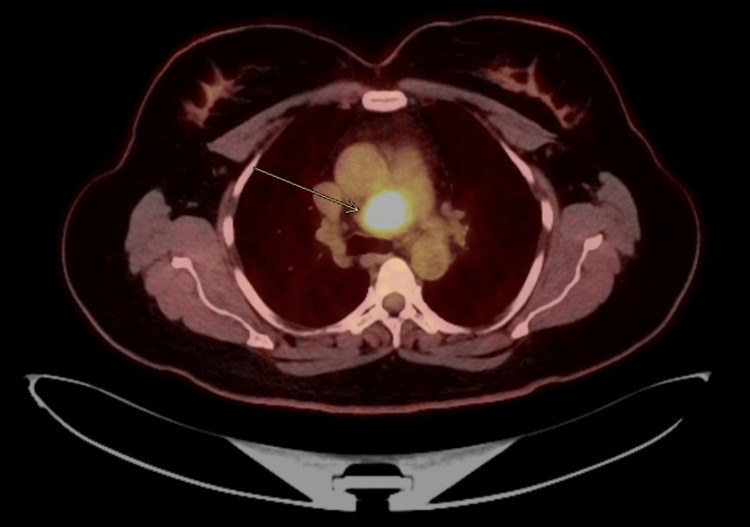
PET-CT scan axial view demonstrating a moderately fluorodeoxyglucose (FDG) avid lower paratracheal mass

The patient reported occasional night sweats and unintentional weight loss but was otherwise asymptomatic. The 24-hour urine metanephrine levels were unremarkable, and serum chromogranin A was slightly elevated. She underwent endobronchial ultrasound (EBUS) with fine need aspiration which was unrevealing due to lack of diagnostic tissue. The patient was referred to thoracic surgery for surgical evaluation. She underwent a positron emission tomography (PET)-CT scan prior to surgery demonstrating a moderately fluorodeoxyglucose (FDG) avid lower paratracheal mass (Figure 3).

**Figure 4:**
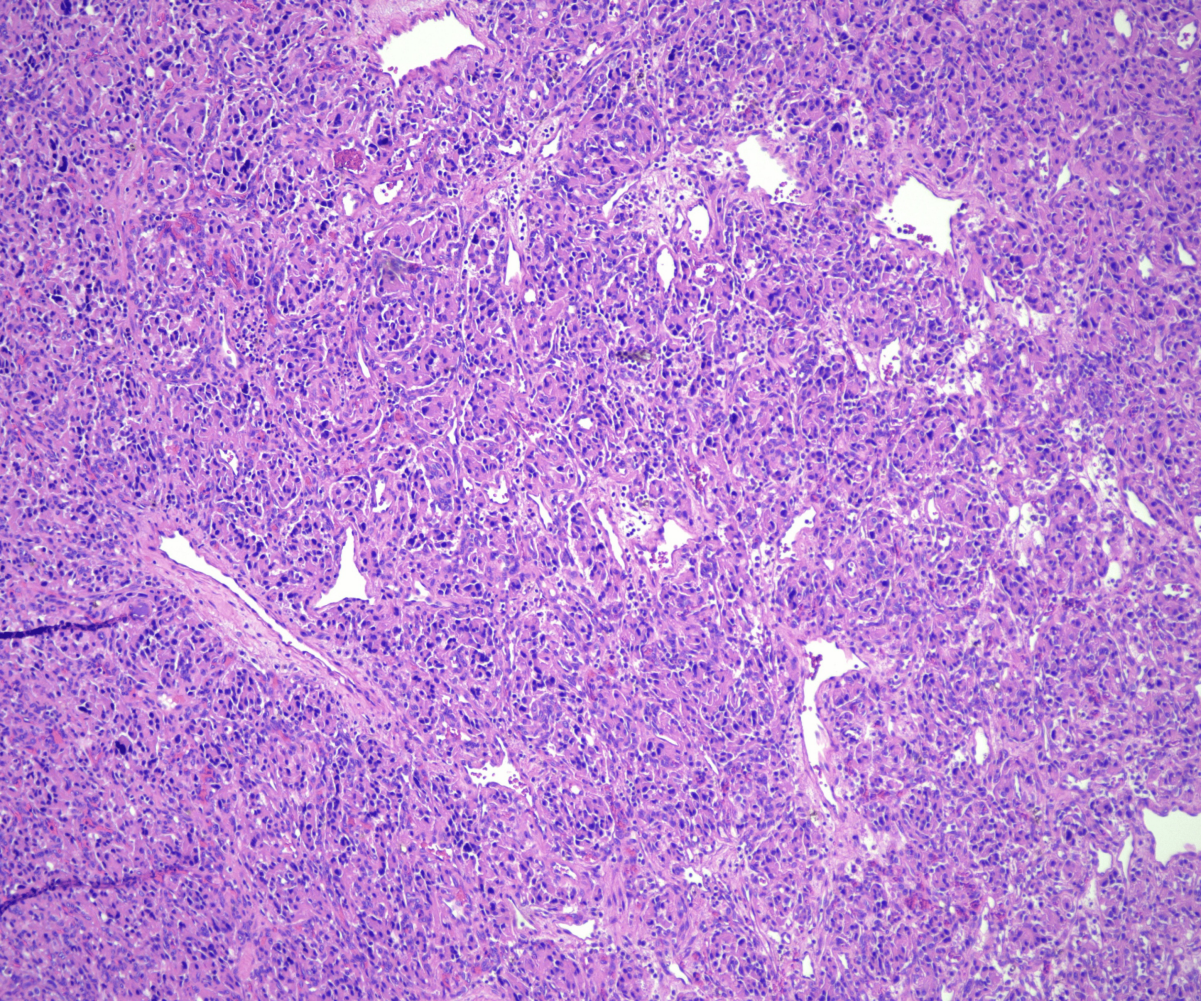
Hemoxylin and eosin staining at 10x magnification showing nests of neoplastic cells in a background of blood vessels, some of which are ectatic

The patient underwent embolization of the feeding vessel prior to surgical resection. The mass was successfully removed and a tissue sample was obtained for biopsy and immunohistochemical staining. Hematoxylin and eosin staining of the tissue at 10x magnification showed nests of neoplastic cells in a background of abundant blood vessels, some of which were ectatic (Figure 4).

**Figure 5:**
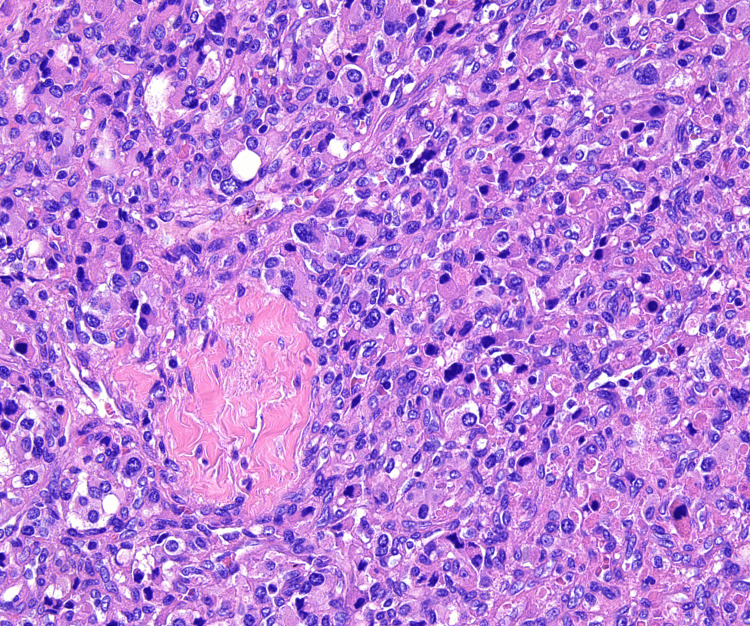
Hematoxylin and eosin staining of the tissue at 40x magnification showing neoplastic cells containing eosinophilic cytoplasm with round nuclei and occasional macronuclei. Scattered nuclear pleomorphism and multinucleation present without significant mitotic activity

At 40x magnification, neoplastic cells containing eosinophilic cytoplasm with round nuclei and occasional macronuclei were observed. Scattered nuclear pleomorphism and multinucleation were present without significant mitotic activity (Figure 5).

**Figure 6:**
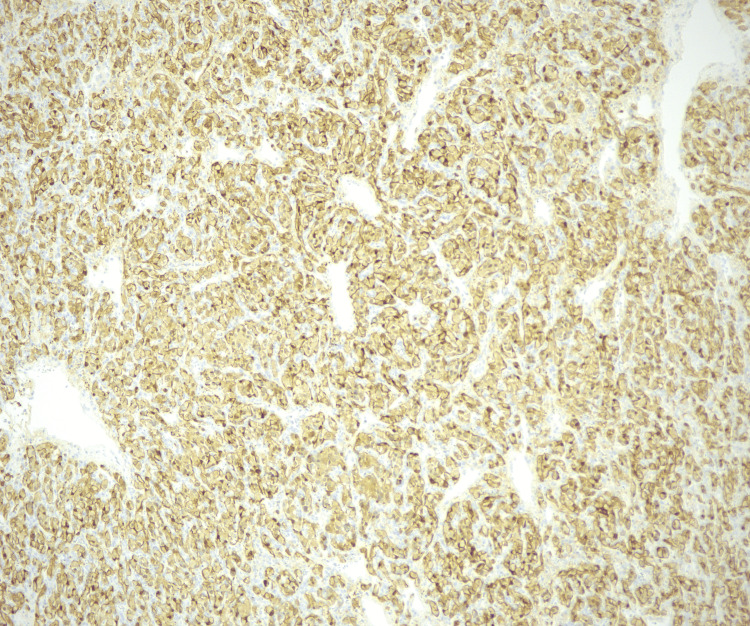
S-100 staining at 10x magnification demonstrating positivity in the sustentacular cells highlighting the presence of an extensive sustentacular cell network

S-100 staining was obtained demonstrating positivity in the sustentacular cells highlighting the presence of an extensive sustentacular cell network (Figure 6). SOX10 staining was also positive in the sustentacular cells. The neoplastic cells in the sample were positive for synaptophysin, chromogranin, retained fumarate hydratase (FH), GATA3, and succinate dehydrogenase complex iron subunit B (SDHB) and negative for cytokeratin (CAM) 5.2, pan-tropomyosin receptor kinase (TRK), and CD61. The Ki-67 index of the neoplastic cells was approximately 3-5%. These findings lead to the diagnosis of paraganglioma.

**Figure 7:**
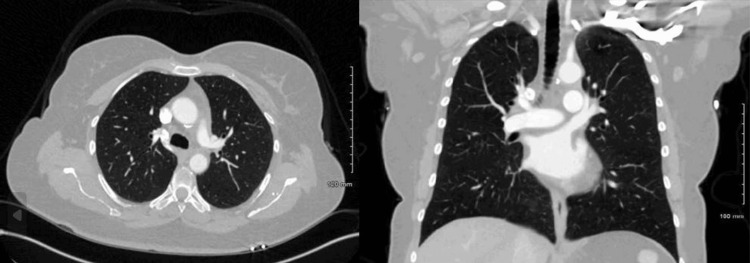
Axial and coronal views of CT chest with contrast approximately one year after resection demonstrating no evidence of disease recurrence

After formal diagnosis and surgical resection, PET-CT was performed showing no evidence of further disease. Repeat CT of the chest with contrast approximately one year after diagnosis showed no evidence of recurrence (Figure 7). The patient will continue to undergo CT scans of the chest every six months to monitor for tumor recurrence. 

## Discussion

Middle mediastinal paragangliomas are a rare presentation of an already rare disease. Extra-adrenal paragangliomas arise from sympathetic or parasympathetic ganglia and are histologically similar to intra-adrenal paragangliomas, commonly called pheochromocytomas [1]. Paragangliomas may arise anywhere ganglia are present, and the location of the incidence varies by ganglia origin. Sympathetic paragangliomas are often located in abdominopelvic regions and may be biochemically active with elevated levels of plasma metanephrines and chromogranin A, resulting in symptoms of catecholamine excess such as diaphoresis, palpitations, and headaches. Serum and urine metanephrine and catecholamine levels should be checked in patients experiencing these symptoms. Parasympathetic paragangliomas are primarily found in the neck and skull base. These lesions are generally non-functional and asymptomatic [1,2].

Infrequently, paragangliomas may be located in the mediastinum. Mediastinal paragangliomas account for less than 2% of all paragangliomas and less than 0.3% of mediastinal masses, with even fewer located in the middle mediastinum [2]. Paragangliomas most often occur sporadically but may be associated with hereditary syndromes such as von Hippel-Lindau, Multiple Endocrine Neoplasia (MEN) syndromes, Neurofibromatosis type 1, and Carney-Stratakis syndrome [3]. Paragangliomas are associated with mutations in succinate dehydrogenase genes. Mutations of the succinate dehydrogenase complex iron sulfur subunit B (SDHB) gene are associated with aggressive clinical disease course [4].

Definitive diagnosis is typically confirmed with histopathology and immunohistochemistry after surgical resection [1,2,5]. Paragangliomas often display a nested growth pattern of chief cells with abundant, basophilic cytoplasm referred to as a zellballen pattern. The tumor cells are typically surrounded by a network of thin blood vessels and supporting cells, also known as sustentacular cells, which are only recognizable if immunohistochemical stains are applied such as S100 or SOX10. These cells are strongly positive for neuroendocrine markers such as chromogranin A and synaptophysin. Other markers such as tyrosine hydroxylase may be present as well. Keratin expression is almost always absent [5,6].

Paragangliomas have a high rate of recurrence which may be delayed until decades after resection. It is recommended that all patients follow up for at least 10 years after resection. Patients with high-risk features such as confirmed germline mutation, young age of onset (<20 years old), and patients carrying the SDHB gene mutation are recommended lifelong follow-up [7,8].

## Conclusions

Paragangliomas are rare neuroendocrine tumors that may arise from sympathetic or parasympathetic ganglia. Paragangliomas may cause symptoms secondary to excessive sympathetic activity from catecholamine secretion or symptoms related to mass effect. Although very rarely found in the mediastinum, paragangliomas are important to consider in the differential diagnosis of mediastinal masses. Succinate dehydrogenase complex iron sulfur subunit B (SDHB) gene mutations are associated with aggressive clinical disease course. Although surgical resection has curative potential, patients require long-term follow-up as these tumors have the potential to recur.

## References

[1] Ikram A, Rehman A (2024). Paraganglioma. StatPearls.

[2] De Palma A, Lorusso M, Di Gennaro F (2018). Pulmonary and mediastinal paragangliomas: rare endothoracic malignancies with challenging diagnosis and treatment. J Thorac Dis.

[3] Zhikrivetskaya SO, Snezhkina AV, Zaretsky AR (2017). Molecular markers of paragangliomas/pheochromocytomas. Oncotarget.

[4] Jochmanova I, Wolf KI, King KS (2017). SDHB-related pheochromocytoma and paraganglioma penetrance and genotype-phenotype correlations. J Cancer Res Clin Oncol.

[5] Asa SL, Ezzat S, Mete O (2018). The diagnosis and clinical significance of paragangliomas in unusual locations. J Clin Med.

[6] Juhlin CC (2021). Challenges in paragangliomas and pheochromocytomas: from histology to molecular immunohistochemistry. Endocr Pathol.

[7] Li M, Prodanov T, Meuter L (2023). Recurrent disease in patients with sporadic pheochromocytoma and paraganglioma. J Clin Endocrinol Metab.

[8] Aygun N, Uludag M (2020). Pheochromocytoma and paraganglioma: from treatment to follow-up. Sisli Etfal Hastan Tip Bul.

